# Anti-inflammatory Diet In Rheumatoid Arthritis (ADIRA)—a randomized, controlled crossover trial indicating effects on disease activity

**DOI:** 10.1093/ajcn/nqaa019

**Published:** 2020-02-13

**Authors:** Anna K E Vadell, Linnea Bärebring, Erik Hulander, Inger Gjertsson, Helen M Lindqvist, Anna Winkvist

**Affiliations:** 1 Department of Internal Medicine and Clinical Nutrition, Institute of Medicine, Sahlgrenska Academy, University of Gothenburg, Gothenburg, Sweden; 2 Department of Rheumatology and Inflammation Research, Institute of Medicine, Sahlgrenska Academy, University of Gothenburg, Gothenburg, Sweden

**Keywords:** anti-inflammatory, rheumatoid arthritis, diet, inflammation, omega-3 fatty acids, probiotics, dietary fiber, DAS28, Sweden

## Abstract

**Background:**

Many patients with rheumatoid arthritis (RA) report symptom relief from certain foods. Earlier research indicates positive effects of food and food components on clinical outcomes in RA, but insufficient evidence exists to provide specific dietary advice. Food components may interact but studies evaluating combined effects are lacking.

**Objectives:**

We aimed to investigate if an anti-inflammatory diet reduces disease activity in patients with RA.

**Methods:**

In this single-blinded crossover trial, 50 patients with RA were randomly assigned to an intervention diet containing a portfolio of suggested anti-inflammatory foods, or a control diet similar to the general dietary intake in Sweden, for 10 wk. After a 4-mo washout period the participants switched diet. Food equivalent to ∼50% of energy requirements was delivered weekly to their homes. For the remaining meals, they were encouraged to consume the same type of foods as the ones provided during each diet. Primary outcome was change in Disease Activity Score in 28 joints-Erythrocyte Sedimentation Rate (DAS28-ESR). Secondary outcomes were changes in the components of DAS28-ESR (tender and swollen joints, ESR, and visual analog scale for general health) and DAS28-C-reactive protein.

**Results:**

In the main analysis, a linear mixed ANCOVA model including the 47 participants completing ≥1 diet period, there was no significant difference in DAS28-ESR between the intervention and control periods (*P* = 0.116). However, in unadjusted analyses, DAS28-ESR significantly decreased during the intervention period and was significantly lower after the intervention than after the control period in the participants who completed both periods (*n* = 44; median: 3.05; IQR: 2.41, 3.79 compared with median: 3.27; IQR: 2.69, 4.28; *P* = 0.04, Wilcoxon's Signed Rank test). No significant differences in the components were observed.

**Conclusions:**

This trial indicates positive effects of a proposed anti-inflammatory diet on disease activity in patients with RA. Additional studies are required to determine if this diet can cause clinically relevant improvements.

This trial was registered at clinicaltrials.gov as NCT02941055.

## Introduction

Rheumatoid arthritis (RA) is a chronic autoimmune disease characterized by synovial inflammation often followed by cartilage and bone erosion (1). The patients suffer from reduced functional ability, pain, and stiffness that often lead to impaired quality of life (2, 3). The prevalence of RA is ∼0.5–1% of the Western population (4) and the disease is more common among women and elderly (5). The current treatment is based on immunosuppression that aims to prevent further joint destruction, reduce the symptoms, and achieve remission (6). However, many do not reach sustained remission (7), and even if disease activity is reduced, many still suffer from disabling symptoms including pain and fatigue (8, 9).

Patients with RA often ask their physician for specific dietary advice and many report that different food items improve or worsen the disease symptoms (10–12). Red meat, alcohol, and soft drinks are examples of foods reported to worsen symptoms, whereas fish and berries are reported to improve symptoms (11, 12). There are few studies investigating whole diets, but beneficial effects on disease activity have been noted in intervention studies with a Mediterranean diet (13) as well as with fasting followed by a vegetarian diet (14) and gluten-free vegan diet (15). Research on the effects of food components on inflammation and patient-perceived symptoms includes n–3 fatty acids, probiotics, vitamin D, and antioxidants. n–3 Fatty acids seem to have positive effects on several outcomes such as erythrocyte sedimentation rate (ESR) and tender joint count (16), and a number of trials using probiotics have shown positive effects on disease activity (17). There seems to be potential for vitamin D as well to reduce disease activity, but very few studies have been performed (18). Further, several antioxidants and sources of bioactive compounds with antioxidative effects have been evaluated and some studies have obtained a reduction in symptoms as well as lower disease activity with these foods and supplements (19–25). Many of these dietary interventions in RA had small study populations and suffered from a poor design. Nevertheless, they indicate that several foods and food components have potential to reduce RA disease activity by lowering grade of inflammation or alleviating symptoms like joint pain. Still, studies investigating the effects of a comprehensive anti-inflammatory portfolio diet are lacking for RA. A portfolio diet contains a combination of foods that may potentiate each other's health effects and there is a need for high-quality studies investigating whether a portfolio diet with anti-inflammatory foods can complement the pharmacological treatment of RA and reduce symptoms further.

The aim of the ADIRA (Anti-inflammatory Diet in Rheumatoid Arthritis) trial (NCT02941055) was to investigate if an anti-inflammatory portfolio diet, rich in n–3 fatty acids, dietary fibers, and probiotics, compared with a control diet nutritionally similar to a typical Swedish diet high in SFAs, but with proportions of protein, total fat, and carbohydrates in line with recommendations, can act as an adjuvant therapy and lower disease activity in patients with RA.

## Methods

### Study participants and study design

Details about recruitment have been described elsewhere (26). In brief, an invitation letter was sent to all participants in the Swedish Rheumatology Quality Register (SRQ) with disease duration ≥2 y, 18–75 y old, and living in the Gothenburg region in Sweden (to be able to receive trial food and attend study visits at the Clinic of Rheumatology, Sahlgrenska University Hospital, Gothenburg). Patients who were interested in participating were invited to a screening visit. Inclusion criteria were Disease Activity Score in 28 joints-Erythrocyte Sedimentation Rate (DAS28-ESR) ≥2.6 (rounded to 1 decimal place) at screening and clinically stable disease under adequate control, i.e., no changes in immunosuppressive treatment [disease-modifying antirheumatic drugs (DMARDs)] during the preceding 8 wk. Exclusion criteria were allergies or intolerances to food included in the study, inability to understand the information, other serious illnesses, pregnancy, and lactation. For practical reasons, ADIRA was performed in 2 batches: February–December 2017 and August 2017–May 2018.

Details about study design have been described previously (26). In this single-blinded controlled crossover trial, participants were allocated (1:1) by a computer-generated randomization list to start with either the intervention diet or control diet followed by a washout period and thereafter the diet regimen was switched. The median (min–max) duration of each diet period was 10 (8–13) wk and the washout period was 4 (2–5) mo. Participants received food to prepare breakfast, 1 snack, and 1 main dish per day for 5 d/wk, providing ∼1100 kcal/d. The food was delivered weekly to their homes by a home delivery food chain at a day and time of their choice. At enrollment, the participants were instructed to abstain from nutritional supplements during the study except those prescribed by a physician.

### Diets

The intervention diet was a portfolio diet combining foods with suggested anti-inflammatory effects and food components with promising effects on RA disease activity and symptoms. **Table 1** describes the foods included in detail. In brief, the main meals in the diet contained fish (mainly salmon) 3–4 times/wk and vegetarian dishes with legumes 1–2 times/wk. Potatoes, whole-grain cereals, vegetables, yoghurt for sauces, spices, and other flavorings were also included. Snacks were composed of fruits, whereas breakfasts contained low-fat dairy, whole-grain cereals, pomegranate and blueberries, nuts, and juice shots with probiotics. The probiotic shot used contained *Lactobacillus plantarum 299v* and was provided to the participants 5 d/wk. For the meals not provided, the participants were instructed to limit their intake of meat to ≤3 times/wk, to eat ≥5 portions/d of fruit, berries, and vegetables (including those provided), to use oil or margarine for cooking, and to choose low-fat dairy and whole-grain cereals.

**TABLE 1:** The anti-inflammatory portfolio diet in the randomized crossover ADIRA (Anti-inflammatory Diet in Rheumatoid Arthritis) trial^1^

	Foods	Comments
Breakfast	Low-fat fermented dairy	
	Granola containing nuts, seeds, and berries	
	Fiber-enriched oats	
	Low-fat milk	
	Walnuts	
	Blueberries/pomegranate	
	Probiotic shot	Juice
Main meal	Salmon	
	Salmon + cod	Ready-to-eat meal: patties (mixed with cream and egg) served with shellfish sauce (cream and wine), soybeans, and peas
	Beans, chickpeas, and/or lentils	Ready-to-eat meals: stew with potatoes and vegetables; stew with bulgur, coconut milk, and vegetables; or patties with vegetables
	Wheat berries	
	Bulgur, whole grain	
	Potatoes	Cooked or oven-baked
	Pasta, whole grain	
	Yogurt (10% fat)	
	Crème fraiche (13% fat)	
	Vegetables (e.g., soy beans, spinach, onion, garlic, pepper)	Large amount
	Mango	
	Rapeseed oil	
	Rice vinegar	
	Soy sauce	
	Honey	
	Spices	
	Lime, lemon	
Snack	Bananas/apples/pears	Two daily

1 The participants were provided with foods corresponding to ∼50% of their daily intake, 5 d/wk. For the remaining intake, they were instructed to consume similar foods to those provided.

The total dietary intake during control periods was intended to correspond nutritionally to the average dietary intake in 45- to 64-y-old men and women in Sweden; 17 energy percent (E%) protein, 34 E% total fat, 13 E% SFAs, and 43 E% carbohydrates (27). The control diet provided by the study contained meat or chicken and refined grains daily, protein bar or quark for snacks, and breakfasts based on either white bread with a butter-based spread and cheese, or a mix of quark and yoghurt with corn flakes and orange juice. Beyond this, the participants were also instructed to consume meat ≥5 times/wk; ≤5 portions/d of fruit, berries, and vegetables; seafood ≤1 time/wk; use butter for cooking; choose high-fat dairy; and avoid products with probiotics. Before each diet period, the participants received a binder including weekly menus and recipes, and instructions on dietary intake for the meals not provided. Three weekly menus were repeated throughout the diet periods.

The macronutrient content of both diets has been described in detail elsewhere (26). In brief, the diets were matched on energy and carbohydrate content, but the intervention diet contained a higher proportion of total fat and unsaturated fat and a lower proportion of saturated fat than the control diet. In contrast, the control diet contained a higher proportion of protein. The fiber as well as the n–3 fatty acids content was considerably higher in the intervention diet, 24 g/d (5.2 g/MJ) and 4 g/d (3 E%), compared with 8.3 g/d (1.8 g/MJ) and 0.8 g/d (0.6 E%) in the control diet, respectively. In an effort to mask the diets to participants, the intervention diet was referred to as “Fiber diet” and the control diet as “Protein diet.” It was communicated to participants that the intention was to study the possible effects of 2 different diets.

Compliance to the diets was assessed through interviews by telephone once mid-period. The participants were asked whether *nothing, part of*, or *all of* the breakfasts, main dishes, and snacks received the week before, was consumed. The telephone call also provided an opportunity for the participants to ask questions about the diet (exchange of food items, preparation, food delivery, etc.) and to report any adverse effects. A scoring system was developed to quantify compliance. All food items in a meal consumed equaled 2 points, parts of the meal 1 point, and nothing consumed 0 points. This yielded a maximum of 30 points and 24 points (80%) was considered good compliance. Because the meals were not adjusted to individual energy needs, maximum points could be given for smaller portions than specified in the menus.

### Dietary assessments

#### 3-d food record

The participants completed a 3-d food record at the end of each period. The food record was to be completed during 3 consecutive days: either Thursday, Friday, and Saturday or Sunday, Monday, and Tuesday. The participants were instructed to perform the food record on identical days on both occasions. They were carefully instructed by a dietitian to preferably weigh all items consumed (except tap water), or if not convenient, to use household measuring spoons and cups. If unable to do so, they estimated the amounts with help from pictures. The participants were also asked to note details such as type of fish and vegetables or fat content of dairy. At the study visits, the dietitian reviewed the food record together with the participant. A booklet with pictures of meals in different sizes (28) was used to aid the participants to estimate the portion sizes for meals where scales or measuring cups had not been used.

The food records were all analyzed by the same dietitian in Dietist Net Pro version 18.12.16 (Kost och Näringsdata AB) using The Swedish Food Composition Database 2017-12-15. If certain food items were not available in this database, the database Fineli 2018-02-28 was used; this was only the case for 8 food items.

#### FFQ

At the screening visit the participants answered an FFQ, with questions about 53 food items reflecting the past 12 mo. The questions only contained frequencies and no quantities, with 1 exception: bread. From this FFQ, dietary quality was acquired through an adapted dietary quality index created by the Swedish National Food Agency (29). An index score of 0–4 points was considered a poor dietary quality, 5–8 points a fair dietary quality, and 9–12 points a high dietary quality (30).

### Lifestyle questionnaire

At screening, the participants filled out a lifestyle questionnaire. They were asked about their highest educational level [primary school (a total of 9 y), 2-y upper secondary school (a total of 11 y), 3-y upper secondary school (a total of 12 y), university degree or equal, or no education], occupational status (does not work, <15 h/wk, 16–30 h/wk, 31–40 h/wk, or >40 h/wk), parents’ birthplace (Europe, Middle East, Africa, Asia, North America, or South America), and cigarette smoking. For description of the study population, 2-y and 3-y upper secondary school were combined into 1 category (“Upper secondary school”). Working <15 and 16–30 h/wk were defined as “Working part time” and 31–40 and >40 h/wk as “Working full time.”

### Outcome variables

#### Disease activity

The composite scores DAS28-ESR and DAS28 using C-reactive protein (CRP) include the numbers of tender and swollen joints out of 28 joints, the patient's estimation of his/her general health on a visual analog scale (VAS-GH), and either ESR or CRP (31). The primary outcome was change in DAS28-ESR and secondary outcomes were changes in the separate components of DAS28-ESR and changes in DAS28-CRP. To enable calculation of DAS28 at the screening visit, participants provided blood samples for ESR and CRP within 1 wk before the visit. For the other visits, fasting blood samples were collected before and directly after each diet period. The samples were immediately transported for routine analysis at the laboratory for Clinical Chemistry at Sahlgrenska University Hospital. Two specially trained research nurses at the Clinical Rheumatology Research Centre, Clinic of Rheumatology, blinded to the treatment, performed the examinations of the joints. DAS28-ESR was calculated as 0.56 × √(Tender joint count) + 0.28 × √(Swollen joint count) + 0.7 × ln ESR + 0.014 × VAS-GH (31). DAS28-CRP was calculated as 0.56 × √(Tender joint count) + 0.28 × √(Swollen joint count) + 0.36 × ln (CRP + 1) + 0.014 × VAS-GH + 0.96 (31). To examine how well participants responded to the diet treatment, European League Against Rheumatism (EULAR) response criteria for clinical trials were used (32). No response is defined as a reduction in DAS28-ESR of ≤0.6 units, or >0.6 to ≤1.2 units combined with DAS28 > 5.1 after the treatment. Moderate response is defined as a reduction of >0.6 to ≤1.2 units in combination with DAS28 ≤ 5.1 or a reduction of >1.2 units in combination with DAS28 > 3.2. Finally, a good response is defined as a reduction of >1.2 units in combination with a DAS28 ≤ 3.2.

#### Other clinical assessments

At screening, height was measured to the closest 0.5 cm with a wall-mounted stadiometer, without shoes. At all visits, weight was measured and BMI was calculated as kg/m^2^. Participants were weighed in light clothing without shoes, and 1 kg was subtracted from the measured weight to account for the clothes. If the participant had difficulties removing shoes such that they remained on, 1.5 kg was subtracted. Participants were encouraged to remain weight-stable throughout the study.

During the intervention and control periods, participants reported any changes in medication or health care visits.

### Ethics

The study was conducted according to the Declaration of Helsinki and approved by the regional ethical review board in Gothenburg (registration number 976-16, November 2016, and supplement T519-17, June 2017). Written and informed consent was provided by all participants.

### Statistics

A sample size of 38 was needed for 90% power to detect a change in DAS28-ESR of 0.6 units (α = 0.05). To account for dropout, 50 participants were recruited.

A linear mixed ANCOVA model was used for the main analysis of DAS28-ESR, ESR, VAS-GH, and DAS28-CRP. Treatment (intervention or control diet), period (first or second of the study's 2 batches), sequence (intervention diet period first or control diet period first), and baseline value for the variable were included as fixed effects. Subject (each participant) was included as a random effect. Age, sex, BMI at baseline, education, nicotine use (yes/no), and dietary quality at baseline were considered potential confounders and were therefore included as covariates. However, none of these variables exhibited any confounding effects (defined as a change in β-estimate of ≥10%) and they were therefore not included in the main model. Regression residuals of VAS-GH and ESR had skewed distributions and these variables were therefore transformed with the square-root function. Both main analyses and sensitivity analyses were performed with the transformed values.

The variables tender joints and swollen joints were categorized into dichotomous variables, <1 and ≥1 tender/swollen joint, to enable the use of a generalized logistic mixed model as the main analysis. Period, treatment, sequence, baseline value for the variable, and dietary quality were included as fixed effects and subject as a random effect. Dietary quality was included because of confounding effects in these regression models.

As supporting analyses, Wilcoxon's Signed Rank test was used to test median changes over time in DAS28-ESR, ESR, VAS-GH, and DAS28-CRP for the separate diet periods as well as for comparison of post-values between diet treatments. Wilcoxon's Signed Rank test was also used to test weight changes over time in the separate periods and to compare these changes, and to evaluate the dietary intake during the separate diet periods. Chi-square test was used to compare the diets’ effects on tender and swollen joints with more comprehensive categorizations of the variables. The categorizations were based on distribution of the variables at the end of diet periods (tender joints: 0, 1–4, 5–10, 11–20, and ≥21; swollen joints: 0, 1–2, 3–5, 6–10, and ≥11), and changes within each diet period (worse, no change, and better). Chi-square test was also used to compare the diets’ effects on treatment response using EULAR response criteria. To investigate if response to the dietary intervention depended on disease activity or dietary intake before the intervention period, the EULAR response criteria variable was dichotomized into responders and nonresponders and the Mann–Whitney *U* test was used to test the difference in median DAS28-ESR and dietary quality index between the response groups.

For all outcome variables, the following sensitivity analyses were performed: *1*) completers only, *2*) per-protocol analyses including participants considered to have a good compliance only, *3*) per-protocol analyses including participants without changes in DMARDs or glucocorticoids during the study only, and *4*) intention-to-treat-analyses with imputed values when outcome data were missing (see details below). The same linear mixed models and generalized logistic mixed models as for the main analyses were used in all these analyses.

Missing values were replaced by the use of 3 different single imputation methods. In the first run, missing post-values were replaced with the median change during the respective periods added to the individual's pre-value for both the control and intervention periods. As a best-case scenario, the same procedure was used for missing data in control periods, but for the intervention periods, the lowest quartile difference was added to the individual's pre-value instead of the median. As a worst-case scenario, the same procedure was used for the control periods, but for the intervention periods, the third quartile difference was added instead of the first. Imputations were performed only for periods with a pre-value, e.g., if a participant dropped out during the first period, no imputations were performed for the intended second period.

Because of the skewed distribution of most variables, all results based on continuous variables are presented as median and IQR unless otherwise stated. Discrete and categorical variables were categorized and are presented as frequency and proportion.

No correction for multiplicity was made and a 5% significance level was used for all the statistical tests. IBM SPSS Statistics version 25 (IBM Corp.) was used for all tests.

## Results

### Subjects and adherence

**FIGURE 1:**
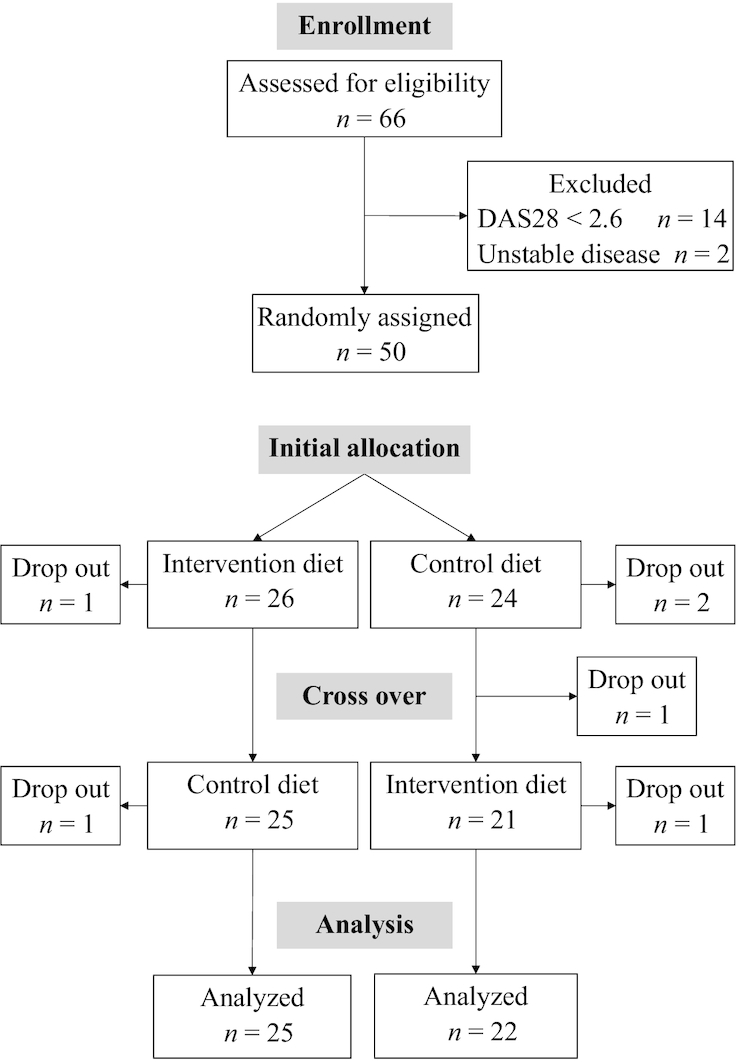
Flowchart of the ADIRA (Anti-inflammatory Diet in Rheumatoid Arthritis) trial. DAS28, Disease Activity Score-28.

A total of 66 patients attended the screening visit and 50 were included in the study. The remaining 16 patients were excluded because of remission (i.e., DAS28 < 2.6) or nonstable disease (**Figure 1**).

Of the 50 included participants, 47 completed ≥1 diet period and 44 completed both diet periods. The reasons for dropout were difficulty eating, onset of other serious illness, and moving outside the food delivery area. There were no indications that the dropouts were due to adverse effects of the study diets. A total of 45 intervention diet periods and 46 control diet periods were completed. Of these, 91% were completed with good compliance: 40 control diets and 43 intervention diets. The 3-d food records displayed several significant differences in nutrient intake between the intervention and control periods (**Table 2**). For example, intakes of fiber, EPA, and DHA were considerably higher during the intervention period (*P* < 0.001). There was no difference in energy intake and weight changes were nonsignificant during the separate diet periods (median: −0.4 kg; IQR: −1.4, 0.6 kg during the intervention period; *P* = 0.082 and median: 0.3 kg; IQR: −0.7, 1.6 kg during the control period; *P* = 0.379), and weight changes were also not significantly different between the diet periods (−0.4 kg compared with 0.3 kg, *P* = 0.122).

**TABLE 2:** Daily dietary intake reported in 3-d food records during intervention periods and control periods by participants in the randomized crossover ADIRA (Anti-inflammatory Diet in Rheumatoid Arthritis) trial^1^

	Intervention^2^ (*n* = 43)	Control^3^ (*n* = 42)	*P* ^4^
Energy, MJ	7.85 [6.48–8.73]	7.27 [6.48–8.68]	0.675
Protein, E%	14 [14–17]	18 [15–20]	0.001
Carbohydrates, E%	41 [36–45]	43 [38–47]	0.468
Fiber, g	24 [20.4–28]	14.3 [11.4–18.2]	<0.001
Total fat, E%	39 [34–42]	36 [32–40]	0.247
SFAs, E%	13 [10–15]	16 [14–17]	<0.001
MUFAs, E%	14 [12–15]	13 [12–14]	0.180
PUFAs, E%	9 [7–10]	4 [4–5]	<0.001
EPA, g	0.55 [0.38–0.87]	0.01 [0.01–0.11]	<0.001
DHA, g	0.90 [0.51–1.39]	0.09 [0.03–0.24]	<0.001
Vitamin C, mg	80 [64–119]	110 [92–143]	0.038
Vitamin D, µg	9.7 [6.9–12.1]	4.4 [2.8–6.4]	<0.001
Vitamin E, mg	13.8 [11.8–16.9]	10.0 [8.2–11.6]	<0.001
Selenium, µg	53 [45–60]	37 [26–54]	0.005
Zinc, mg	7.9 [6.5–10.1]	9.3 [8.1–10.8]	0.006

1 Values are median [IQR]. E%, energy percent.

2 Anti-inflammatory diet.

3 Diet nutritionally similar to a typical Swedish diet.

4 Differences between the diets analyzed with Wilcoxon's Signed Rank test.

**Tables 3** and **4** present the baseline data. The majority (77%) of the participants were women and almost half of the group had a university degree or equivalent (49%). Forty-three percent did not work. Almost one-third (32%) had a BMI corresponding to obesity (BMI ≥ 30). The median (IQR) dietary index score was 6 (5–7) and the majority (79%) had a fair dietary quality at baseline. Median (IQR) DAS28-ESR was 3.7 (3.0–4.6), corresponding to a moderate disease activity (32).

**TABLE 3:** Baseline characteristics of participants in the ADIRA (Anti-inflammatory Diet in Rheumatoid Arthritis) trial who completed ≥1 diet period: anti-inflammatory diet (intervention) and/or a diet nutritionally similar to a typical Swedish diet (control)^1^

	Mean ± SD or *n* (%)	Median [IQR]
Age, y	61 ± 12	63 [54–71]
Female	36 (77)	
Anthropometry		
Weight, kg	77.8 ± 14.3	77.8 [66.9–85.4]
Height, cm	168 ± 9	168 [162–174]
BMI, kg/m^2^	27.6 ± 5.4	26.6 [24–31.8]
Waist circumference, cm	92.0 ± 14.1	92.0 [83–100]
Hip circumference, cm	106.1 ± 11.2	106.0 [98–112]
Parents’ birthplace		
Europe	44 (94)	
Africa	1 (2)	
Asia	2 (4)	
Level of education		
Primary school	8 (17)	
Upper secondary school	16 (34)	
University degree or equivalent	23 (49)	
Occupational status		
Does not work	20 (43)	
Working part time	8 (17)	
Working full time	19 (40)	
Current smokers	2 (4)	
Dietary intake		
Dietary quality index score	6.1 ± 1.9	6 [5–7]
Poor dietary quality	7 (15)	
Fair dietary quality	37 (79)	
High dietary quality	3 (6)	

1 *n* = 47.

**TABLE 4:** The rheumatic disease and treatment at baseline for participants in the ADIRA (Anti-inflammatory Diet in Rheumatoid Arthritis) trial who completed ≥1 diet period: anti-inflammatory diet (intervention) and/or a diet nutritionally similar to a typical Swedish diet (control)^1^

	Mean ± SD or *n* (%)	Median [IQR]
Disease duration, y	20.0 ± 9.5	19.2 [10.6–28.2]
DAS28-ESR	3.8 ± 0.9	3.7 [3.0–4.6]
DAS28-CRP	3.6 ± 0.8	3.5 [2.9–4.1]
Tender joint count (0–28)	4.1 ± 4.2	2.0 [1.0–6.0]
Swollen joint count (0–28)	1.8 ± 1.6	1.0 [1.0–2.0]
ESR, mm	19.2 ± 10.8	20.0 [11.0–26.0]
CRP, mg/L	4.2 ± 4.7	3.0 [1.0–6.0]
VAS-GH, mm	44 ± 22	43 [26–59]
ACPA and/or RF positive	34 (72)	
Drug treatment		
Non-NSAID analgesic	13 (28)	
NSAIDs	24 (51)	
DMARDs	42 (89)	
DMARD anti-TNF	16 (34)	
DMARD methotrexate	31 (66)	
DMARD sulfalazin	6 (13)	
Blood pressure lowering	20 (43)	
Glucocorticoids	12 (26)	

1 *n* = 47. ACPA, anti-citrullinated protein antibodies; CRP, C-reactive protein; DAS28, Disease Activity Score-28; DMARD, disease-modifying antirheumatic drug; ESR, erythrocyte sedimentation rate; NSAID, nonsteroidal anti-inflammatory drug; RF, rheumatoid factor; VAS-GH, visual analog scale for general health.

Adverse effects in the form of upset stomach were reported during 13 (29%) of the intervention periods and 4 (8.7%) of the control periods. Stomach ache, gas, diarrhea, heartburn, and nausea were described as adverse effects during the intervention period. Constipation, bloating, and acid reflux were reported during the control period. However, some adverse effects were present only at the start of a diet period.

### Effect of intervention on disease activity

No significant difference between the intervention and control periods for the primary outcome DAS28-ESR was seen using linear mixed ANCOVA model analysis (**Table 5**) (mean: −0.289; 95% CI: −0.652, 0.075; *P* = 0.116). However, DAS28-ESR was significantly lower after the intervention period than before (**Table 6**) (*P* = 0.012, Wilcoxon's Signed Rank test). In contrast, no differences were found before and after the control period (*P* = 0.694). In addition, Wilcoxon's Signed Rank test showed a significantly lower DAS28-ESR after the intervention period than after the control period (Table 6) (median: 3.05; IQR: 2.41, 3.79 and median: 3.27; IQR: 2.69, 4.28, respectively; *P* = 0.04).

**TABLE 5:** Modeled estimates of differences in disease activity between an anti-inflammatory diet (intervention) and a diet nutritionally similar to a typical Swedish diet (control) for 10 wk among patients with rheumatoid arthritis in the randomized crossover ADIRA (Anti-inflammatory Diet in Rheumatoid Arthritis) trial^1^

	Intervention	Control	Difference between periods^2^	95% CIs	*P*
DAS28-ESR^3^					
Mean change (95% CIs)	−0.369 (−0.628, −0.111)	−0.080 (−0.335, 0.174)	−0.289	−0.652, 0.075	0.116
Tender joints,^4^ %					
No tender joints at end of period	33.2 (16.1, 56.2)	27.1 (12.7, 48.7)	6.1	−15.2, 27.3	0.572
Swollen joints,^4^ %					
No swollen joints at end of period	48.6 (23.8, 74.1)	37.3 (16.2, 64.5)	11.4	−14.4, 37.2	0.383
ESR^3^,^5^					
Mean change (95% CIs)	−0.051 (−0.347, 0.245)	0.210 (−0.081, 0.501)	−0.261	−0.661, 0.138	0.194
VAS-GH,^3^, ^5^ mm					
Mean change (95% CIs)	−0.219 (−0.742, 0.303)	0.099 (−0.415, 0.613)	−0.319	−0.991, 0.354	0.343
DAS28-CRP^3^					
Mean change (95% CIs)	−0.455 (−0.698, −0.212)	−0.222 (−0.461, 0.017)	−0.233	−0.569, 0.103	0.169

1 Participants completing ≥1 diet period (*n* = 47). CRP, C-reactive protein; DAS28, Disease Activity Score-28; ESR, erythrocyte sedimentation rate; VAS-GH, visual analog scale for general health.

2 Intervention minus control, post-period values.

3 Analyzed by use of a linear mixed model with period, treatment, sequence, and baseline value as fixed effects and subject as random effect.

4 Analyzed by use of a generalized linear mixed model with period, treatment, sequence, baseline value, and dietary quality as fixed effects, subject as random effect, and the outcome variable dichotomous as 0 = no tender/swollen joints, 1 = ≥1 tender/swollen joint.

5 Variable transformed with the square-root function for normal distribution before analysis. The values presented are the transformed values.

**TABLE 6:** Disease activity in patients with rheumatoid arthritis consuming an anti-inflammatory diet (intervention) and diet nutritionally similar to a typical Swedish diet (control) for 10 wk in the randomized crossover ADIRA (Anti-inflammatory Diet in Rheumatoid Arthritis) trial^1^

	Intervention period				Control period				
	Pre (*n* = 46)	Post (*n* = 45)	Δ	*P* ^2^	Pre (*n* = 47)	Post (*n* = 46)	Δ	*P* ^3^	*P*
DAS28-ESR	3.39 [2.66–4.41]	3.05 [2.41–3.79]	−0.42	0.012	3.42 [2.86–4.46]	3.27 [2.76–4.31]	−0.05	0.694	0.040^4^
Tender joints									
0	11 (23.9)	16 (35.6)			8 (17.0)	14 (30.4)			0.565^5^
1–4	20 (43.5)	23 (51.1)			26 (55.3)	22 (47.8)			
5–10	13 (28.3)	5 (11.1)			10 (21.3)	6 (13.0)			
11–20	2 (4.3)	1 (2.2)			3 (6.4)	4 (8.7)			
≥21	0 (0.0)	0 (0.0)			0 (0.0)	0 (0.0)			
Worse		9 (20.0)				14 (30.4)			0.276^5^
No change		11 (24.4)				14 (30.4)			
Better		25 (55.6)				18 (39.1)			
Swollen joints									
0	16 (34.8)	21 (46.7)			14 (29.8)	19 (41.3)			0.893^5^
1–2	21 (45.7)	19 (42.2)			25 (53.2)	23 (50.0)			
3–5	8 (17.4)	4 (8.9)			8 (17.0)	3 (6.5)			
6–10	1 (2.2)	1 (2.2)			0 (0.0)	1 (2.2)			
≥11	0 (0.0)	0 (0.0)			0 (0.0)	0 (0.0)			
Worse		9 (20.0)				6 (13.0)			0.331^5^
No change		14 (31.1)				21 (45.7)			
Better		22 (48.9)				19 (41.3)			
ESR, mm	18 [9–26]	17 [8–29]	0.0	0.984	19 [9–26]	19.5 [8–33]	1.0	0.073	0.155^4^
VAS-GH, mm	40 [21–59]	34 [19–54]	−4.0	0.265	37 [25–59]	44 [25–62]	0.0	0.668	0.080^4^
DAS28-CRP	3.18 [2.40–3.96]	2.76 [2.00–3.40]	−0.47	0.003	3.19 [2.64–4.02]	2.95 [2.26–3.66]	−0.26	0.079	0.107^4^

1 Values are median [IQR] or *n* (%) unless otherwise stated. CRP, C-reactive protein; DAS28, Disease Activity Score-28; ESR, erythrocyte sedimentation rate; VAS-GH, visual analog scale for general health.

2 Wilcoxon's Signed Rank test, comparison of pre-value and post-value for each diet, *n* = 45.

3 Wilcoxon's Signed Rank test, comparison of pre-value and post-value for each diet, *n* = 46.

4 Wilcoxon's Signed Rank test, comparison of post-values between diets, including participants completing both diet periods (*n* = 44). As a consequence, there were small changes in median and/or IQR for the control period and/or intervention period.

5 Chi-square test (post-values).

There were no significant differences between the diet periods for tender and swollen joints using adjusted generalized mixed logistic model analyses (Table 5) or chi-square test. Still, 25 participants (56%) had fewer tender joints after the intervention period compared with 18 participants (39%) after the control period. No difference between the diet periods was obtained for ESR or VAS-GH using adjusted linear mixed-model analyses (Table 5). However, a trend toward a higher ESR after the control period than before was seen with Wilcoxon's Signed Rank test (Table 6) (*P* = 0.073) and there was a trend toward participants more often reporting feeling better using VAS-GH after the intervention period than after the control period (Table 6) (*P* = 0.080).

DAS28-CRP did not differ significantly between the 2 diet periods using linear mixed ANCOVA model analysis (Table 5) (mean: −0.233; 95% CI: −0.569, 0.103; *P* = 0.169). However, Wilcoxon's Signed Rank test showed a significantly lower DAS28-CRP after the intervention period than before (Table 6) (*P* = 0.003) and a trend toward lower DAS28-CRP after the control period than before (*P* = 0.079).

**FIGURE 2:**
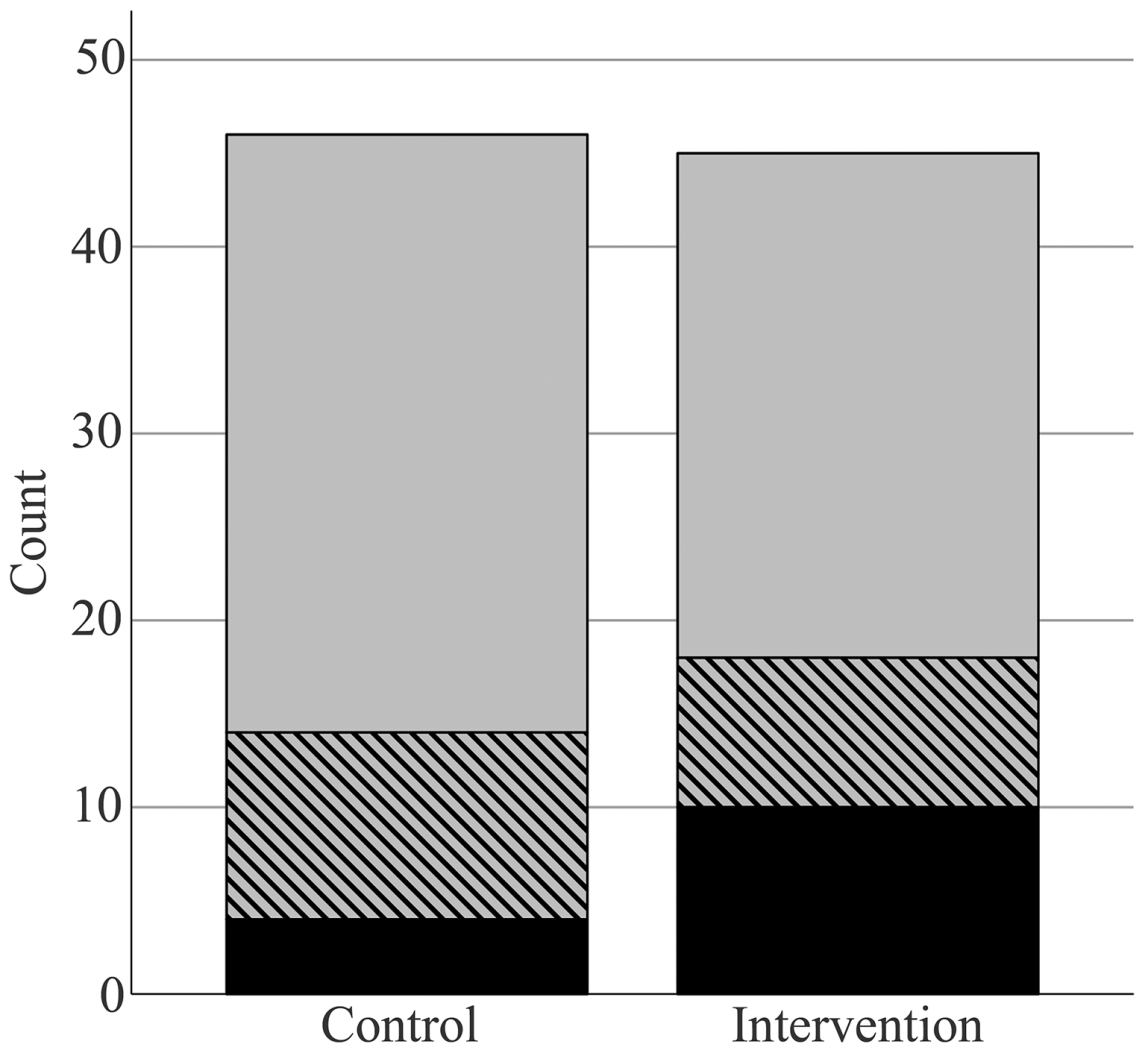
Disease Activity Score-28 European League Against Rheumatism (EULAR) response to an anti-inflammatory diet (intervention, *n* = 45) and a diet nutritionally similar to a typical Swedish diet (control, *n* = 46) in the crossover ADIRA (Anti-inflammatory Diet in Rheumatoid Arthritis) trial. Chi-square test was used to compare the diets’ effects on treatment response and there was no significant difference (*P* = 0.201). No response = grey, moderate response = striped, good response = black.

**FIGURE 3:**
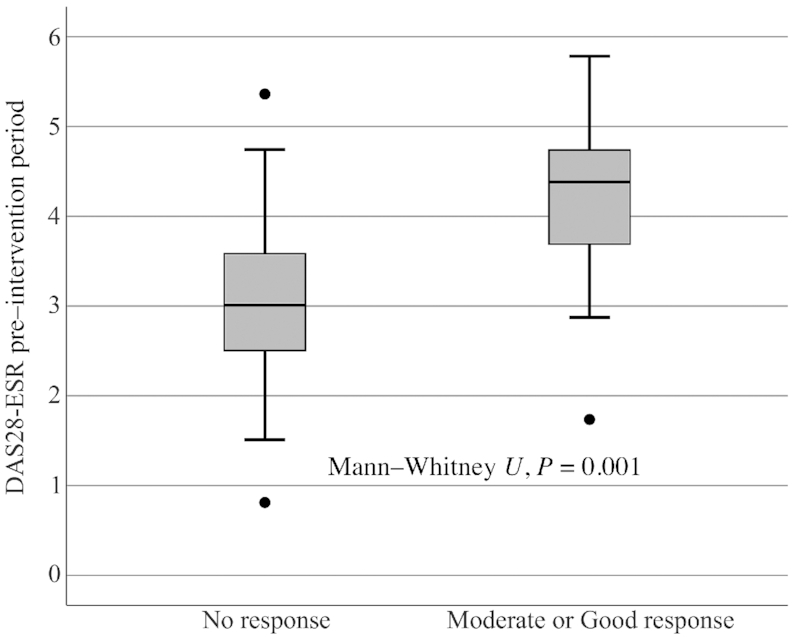
The impact of DAS28-ESR pre–intervention period on treatment response according to European League Against Rheumatism (EULAR) response criteria grouped into 2 categories in the crossover ADIRA (Anti-inflammatory Diet in Rheumatoid Arthritis) trial (*n* = 45). Difference in median DAS28-ESR between responders and nonresponders was tested using the Mann–Whitney *U* test and responders had a higher DAS28-ESR preintervention than nonresponders (*P* = 0.001). DAS28, Disease Activity Score-28; ESR, erythrocyte sedimentation rate.

No significant difference between the intervention and control periods was seen in EULAR response criteria, although 10 participants (22%) had a good response to the intervention diet compared with 4 participants (8.7%) to the control diet (*P* = 0.201) (**Figure 2**). No difference in dietary quality at baseline was seen among nonresponders to the intervention diet compared with responders (*P* = 0.579). Responders had significantly higher DAS28-ESR before the intervention period than nonresponders (*P* = 0.001) (**Figure 3**).

In sensitivity analyses including only participants who completed both diet periods (*n* = 44), the results did not differ from the main analyses (data not shown). When excluding participants with poor compliance, the results did not differ from the main analyses (data not shown), except for DAS28-CRP where there was a significant reduction during the control period as well (mean: −0.318; 95% CI: −0.565, −0.071). When analyzing outcomes only for participants without changes in DMARDs or glucocorticoids (*n* = 25), trends toward differences favoring the intervention diet were seen in DAS28-ESR, VAS-GH, and DAS28-CRP (mean difference: −0.442; 95% CI: −0.917, 0.033; *P* = 0.067; mean difference: −0.700; 95% CI: −1.462, 0.062; *P* = 0.070; and mean difference: −0.407; 95% CI: −0.851, 0.038; *P* = 0.071, respectively). For remaining outcomes, similar null results as in the main analyses were obtained (data not shown). In the intention-to-treat analyses with imputed values for missing data using the best-case scenario, a trend toward a lower DAS28-ESR (**Supplemental Table 1**) (*P* = 0.057) after the intervention period than after the control period was found.

## Discussion

This randomized, single-blinded controlled crossover trial aimed to investigate possible effects on disease activity among patients with RA, of a proposed anti-inflammatory diet containing foods rich in n–3 fatty acids, fiber, antioxidants, and probiotics. In our main analyses, we found no significant difference in effects on DAS28 and its components between this diet and a diet nutritionally similar to a typical Swedish diet. Nevertheless, we did obtain a significant improvement in disease activity during the intervention period and significantly lower DAS28 after intervention than after control in our unadjusted model.

The anti-inflammatory diet used in this crossover trial is similar to the Mediterranean diet used in Sköldstam et al.’s (33) parallel trial and based on unadjusted analyses, the obtained results are consistent; a reduced DAS28 during the intervention period and a larger reduction than during the control period. During our intervention period, we achieved a median reduction of 0.42 units in DAS28, whereas Sköldstam et al. achieved a reduction of 0.56 units. However, disease activity before intervention was higher in Sköldstam et al.: 4.4 compared with 3.4 in our study. It is possible that the DAS28-ESR inclusion criterion in ADIRA (≥2.6) was set too low. Perhaps participants were too healthy to achieve a clinically relevant reduction in disease activity. Indeed, during the intervention period, ADIRA responders according to EULAR response criteria had significantly higher DAS28 pretreatment than nonresponders.

With mixed-model analysis that accounts for intraindividual dependency and missing data, we did not obtain significant differences between the diet periods in disease activity. Still, when imputing results for dropouts as a best-case scenario, we obtained a trend toward a lower DAS28 from the proposed anti-inflammatory diet (*P* = 0.057). This indicates that the result in our main analysis could be due to a too small sample size combined with a lower than expected effect on disease activity.

One component of the ADIRA portfolio diet that distinguishes it from a Mediterranean diet is the probiotics. Patients with RA seem to have a less diverse microbiota composition than healthy individuals (34), and probiotics have the ability to alter the intestinal microbiota and downregulate immune response (35). In clinical studies, several strains of probiotics have been used to ameliorate symptoms in patients with RA (35–38), with conflicting results. Some studies have obtained reduced disease activity with *Lactobacillus casei*, alone or together with *Lactobacillus acidophilus* and *Bifidobacterium bifidum* (35, 38). However, it is not possible to compare ADIRA with this research because the strains used differ.

The beneficial effects of n–3 fatty acids in RA have been studied, and supplementation seems to reduce concentrations of mediators of inflammation (39). A recent systematic review and meta-analysis on effects of PUFAs concluded significant positive effects on several outcomes in RA, although not in DAS28 (16). Some individual trials have shown positive effects of n–3 fatty acids, but these have several limitations (40–43). As an alternative to n–3 supplements ADIRA used fish intake. The intervention diet was intended to be viable in daily life, and therefore did not aim to reach the amounts of EPA and DHA used in supplementation trials. Observational data (44) suggest that consuming fish at least twice per week is associated with lower DAS28-CRP (−0.51), which is further reduced by every additional serving. Moreover, a previous study from our research group showed significant improvements in DAS28-CRP with a mussel diet (low dose of n–3 fatty acids), comparable with the change during the intervention period in ADIRA (45). In that study, EULAR response criteria differed significantly between the control and intervention periods. This was not the case in ADIRA, again perhaps because of the lower disease activity at baseline.

What constitutes an anti-inflammatory diet is ambiguous. A strong public interest in anti-inflammatory food items has recently emerged and foods often mentioned in this context include fish, fermented dairy products, fruits and vegetables, whole grain, and spices (e.g., ginger, turmeric). Even though ADIRA did not provide the same types and large amounts of probiotics and n–3 fatty acids as in previous studies showing anti-inflammatory effects in RA, we obtained some effects on disease activity. Our intervention diet had several other potentially bioactive components: prebiotics from fiber; antioxidants like phytochemicals from fruits and berries, especially pomegranate and blueberries; and vitamin D from both fish and low-fat dairy. The proposition about food and food components potentiating each other in their effects on disease activity still remains to be explored because this was not the main aim of the ADIRA study. Nevertheless, although no differences between the diet periods could be seen in the main analyses, significant improvements were noted during the intervention period and in unadjusted analyses. Also, more participants had a good response to the intervention diet than to the control diet, although the number was not significantly different. With a larger and more comprehensive intervention, i.e., providing the participants with all meals and for a longer period, we might have obtained significant improvements even in our main analyses, closer to the proposed clinical relevance of 0.6 units in DAS28. Also, because the majority of ADIRA participants had a fair dietary quality at baseline, perhaps they were unable to receive further positive effects from the intervention diet.

This trial has some limitations. Firstly, to fully blind a whole diet intervention is difficult. We made an effort to mask the intervention to participants and, based on the participants’ reactions and comments, we believe this was successful. Still, some participants had perceptions on healthy diet which could have influenced the subjective study outcomes. Secondly, the participants only received half of their daily intake 5 d/wk. Larger provisions may have yielded a larger effect. On the other hand, this could have resulted in more dropouts, poor compliance, or difficulties in recruitment. In addition, for practical reasons, recruitment took place only in the Gothenburg area, thus possibly affecting generalizability. Although it was difficult to recruit men, the gender distribution in ADIRA (77% women) is comparable with that of the annual incidence of RA in Sweden (70% women) (46). Finally, for ethical reasons we did not restrict pharmacological drug use during the study. Instead, we performed sensitivity analyses with those not making changes in RA-drug use.

This study also has several strengths. The trial has an unusually high quality for a whole diet intervention: a randomized crossover design eliminating several confounding effects, and single-blinded on behalf of assessors with efforts to also mask the intervention to participants. The participants were instructed to not change their background diet, such as intake of coffee, tea, or alcoholic beverages. Because a crossover design implies that every participant is his/her own control, it is unlikely that the background diet would have added to the variability in the results. To minimize possible carryover effects, participants were also instructed to return to their usual dietary habits during the washout period. Participants remained weight-stable, ensuring that the obtained effects did not derive from weight reduction. We used the SRQ for recruitment, meaning that all eligible patients received an invitation. The trial was carried out across all seasons and participants were randomly assigned to which diet to begin with. Hence, possible seasonal symptom fluctuations (47) should not have affected the results. The study showed high compliance, likely because of the home delivery of food, the use of common foods that participants were accustomed to, and the fact that all meals were ready meals or easy to prepare which, owing to the joint destruction, is of importance to this patient group.

In conclusion, in our main analyses in this randomized crossover trial, we did not obtain significant, clinically relevant reductions in DAS28 or its separate components with a portfolio diet containing food items with suggested anti-inflammatory properties compared with a diet nutritionally similar to a typical Swedish diet. However, improvements during the intervention period as well as differences between end results of the 2 diet periods were significant in unadjusted analyses. Hence, this study indicates positive effects of a proposed anti-inflammatory diet as an adjuvant therapy in patients with RA. Additional studies are needed to determine whether this diet can induce relevant improvements in RA disease activity.

## Supplementary Material

nqaa019_Supplemental_File

## Notes

Supported by the Swedish Government under the ALF agreement (grant ALFGBG-716341 to AW), Forskningsrådet för Arbetsliv och Socialvetenskap (grant 2017-00318 to AW), the Lennander Foundation, Sahlgrenska University Hospital Foundations (to LB), the Inger Bendix Foundation (to AW), and the Gothenburg Region Foundation for Rheumatology Research (GSFR) (to LB).

The funders had no role in the design, implementation, analysis, and interpretation of the data.

Supplemental Table 1 is available from the “Supplementary data” link in the online posting of the article and from the same link in the online table of contents at https://academic.oup.com/ajcn/.

Data described in the article will be made available upon reasonable request. Because of Swedish law, data cannot be shared publicly.

Abbreviations used: ADIRA, Anti-inflammatory Diet in Rheumatoid Arthritis; CRP, C-reactive protein; DAS28, Disease Activity Score-28; DMARD, disease-modifying antirheumatic drug; ESR, erythrocyte sedimentation rate; EULAR, European League Against Rheumatism; E%, energy percent; RA, rheumatoid arthritis; SRQ, Swedish Rheumatology Quality Register; VAS-GH, visual analog scale for general health.
