# Supplementary material for: Anti-inflammatory Diet In Rheumatoid Arthritis (ADIRA)—a randomized, controlled crossover trial indicating effects on disease activity
Source: Am J Clin Nutr. 2020 Feb 13;111(6):1203–13. doi: 10.1093/ajcn/nqaa019 (PMC7266686; doi:10.1093/ajcn/nqaa019)
Supplement: nqaa019_Supplemental_File [file nqaa019_supplemental_file.pdf]

Anti-inflammatory diet in rheumatoid arthritis (ADIRA) - a randomized, controlled crossover trial indicating effects on disease activity (Vadell, A et al.) Online Supplementary Material.

Supplementary Table 1 Modelled estimates of differences in disease activity among patients with RA between end of diet periods (anti-inflammatory diet as intervention and a diet nutritionally similar to a typical Swedish diet as control) in the ADIRA trial, with imputed values for missing data.[1]

	Q2[2]		Q1 (best-case scenario)[3]		Q3 (worst-case scenario)[4]	
	Difference (95% CIs)	*P*-value	Difference (95% CIs)	*P*-value	Difference (95% CIs)	*P*-value
DAS28-ESR[5]	-0.301 (-0.648, 0.046)	0.087	-0.339 (-0.689, 0.011)	0.057	-0.282 (-0.630, 0.067)	0.111
Tender joints (%)[6]	3.4 (-16.8, 23.7)	0.735	5.8 (-14.8, 26.3)	0.577	3.4 (-16.8, 23.7)	0.735
Swollen joints (%)[6]	9.1 (-15.9, 34.1)	0.473	12.2 (-12.8, 37.2)	0.335	9.1 (-15.9, 34.1)	0.473
ESR[5,7]	-0.258 (-0.636, 0.120)	0.175	-0.302 (-0.686, 0.082)	0.120	-0.226 (-0.607, 0.155)	0.238
VAS-GH (mm)[5,7]	-0.269 (-0.908, 0.370)	0.401	-0.290 (-0.930, 0.350)	0.365	-0.233 (-0.874, 0.407)	0.466
DAS28-CRP[5]	-0.241 (-0.562, 0.079)	0.137	-0.267 (-0.590, 0.055)	0.102	-0.216 (-0.539, 0.106)	0.184

[1]Difference=Intervention−Control. Imputed values for three control periods and two intervention periods, and a total of 50 participants included in the analyses. ADIRA, Anti-inflammatory diet in Rheumatoid Arthritis; CRP, C-reactive protein, DAS28, Disease Activity Score-28; ESR, Erythrocyte sedimentation rate; VAS-GH, Visual Analogue Scale for General health

[2]Missing post-values replaced with period-specific median difference added to the participants' pre-value.

[3]Missing post-values replaced with median difference of control periods added to the participants' pre-value if control period, or lower quartile difference of intervention periods if intervention period.

[4]Missing post-values replaced with median difference of control periods added to the participants' pre-value if control period or third quartile difference of intervention periods if intervention period.

[5]Analyzed by use of a linear mixed model with period, treatment, sequence and baseline value as fixed effects and subject as random effect.

[6]Analyzed by use of a generalized linear mixed model with period, treatment, sequence, baseline value and dietary quality as fixed effects and subject as random effect, and the outcome variable dichotomous as 0=no tender/swollen joints, 1= ≥1 tender/swollen joint.

[7]Variable transformed with square root function for normal distribution before analysis. The values presented are the transformed values.
